# CYP51 Paralogue Structure Is Associated with Intrinsic Azole Resistance in Fungi

**DOI:** 10.1128/mBio.01945-21

**Published:** 2021-10-05

**Authors:** N. Van Rhijn, M. Bromley, M. Richardson, P. Bowyer

**Affiliations:** a Manchester Fungal Infection Group, Division of Infection, Immunity and Respiratory Medicine, The University of Manchester, Manchester, United Kingdom; b NHS Mycology Reference Centre Manchester, ECMM Centre of Excellence, Wythenshawe Hospital, Manchester University NHS Foundation Trust, Manchester, United Kingdom; Duke University Medical Center; Universidad de Córdoba

**Keywords:** *Aspergillus*, CYP51, *Penicillium*, antifungal agents, antifungal resistance, cryptic, paralogy

## Abstract

Azoles are the most commonly used clinical antifungal therapy and also play an important role in control of plant pathogens. Intrinsic resistance to the azole class of fungicides, which target lanosterol demethylase (CYP51), is observed in many fungal species; however, the mechanisms underpinning this phenomenon are unknown. In this study, 5 azole-resistant *Penicillium* isolates from patients attending the UK National Aspergillosis Centre that could not be morphologically identified to species level were analyzed by genome sequencing. The genomes and CYP51 paralogue structure from these isolates were compared with those of 46 representative fungal isolates to identify to species level and examine possible mechanisms of drug resistance. Analysis of CYP51 paralogues showed that azole-resistant isolates from this study (*n* = 2) and from public databases (*n* = 6) contained a new CYP51 paralogue, CYP51D, which was associated with azole resistance in 6/8 cases and never occurred in azole-sensitive species (46/46 tested). Furthermore, one isolate from this study and an azole-resistant Aspergillus fumigatiaffinis isolate were shown to encode a CYP51A paralogue, CYP51A2. Introduction of CYP51A2 to the closely related but azole-sensitive Aspergillus fumigatus resulted in azole resistance. The identification of novel CYP51A and CYP51D paralogues in resistant fungi and the observation that resistance to azoles can be conferred by introducing a CYP51A paralogue from a resistant species into an azole-sensitive species are a potentially important new azole resistance mechanism.

## OBSERVATION

Fungi are a major cause of morbidity and mortality in humans and economically cause damaging crop loss in agriculture. One of the main methods to reduce fungal burden is treatment with azole antifungals which act by inhibiting CYP51 enzyme activity in the sterol biosynthesis pathway. In recent years resistance to azoles has become commonly reported and is an increasing concern in clinical and agricultural practice (1–3). Two main forms of resistance to azoles are observed. The first involves acquisition of mutations in the fungal genome which may include the target CYP51 enzyme, efflux pumps (4), or transcription factors regulating their function (5), and the second involves intrinsic resistance or insensitivity to the drug observed as a characteristic of certain species. For example, many filamentous fungi are intrinsically resistant to fluconazole due to the T301I substitution in the CYP51A protein (6, 7). This second form of azole insensitivity occurs in many species of clinical fungal pathogens and those of agricultural importance, and understanding this phenomenon is important in developing strategies to identify and counteract azole insensitivity.

The mechanism of intrinsic resistance or insensitivity in such species is not well understood. Most mold fungal species carry two CYP51 paralogues, CYP51A and -B. Despite this, the vast majority are azole sensitive. In the intrinsically resistant Fusarium graminearum, there are 3 CYP51 paralogues; however, the CYP51C paralogue appears to play no role in ergosterol biosynthesis or azole resistance (8–10). The rationale of the study was to use whole-genome sequencing to precisely define the species and to examine whether CYP51 duplication or paralogy could form the basis for an azole resistance mechanism in these fungi.

### CYP51 paralogue distribution correlates with azole resistance.

**FIG 1:**
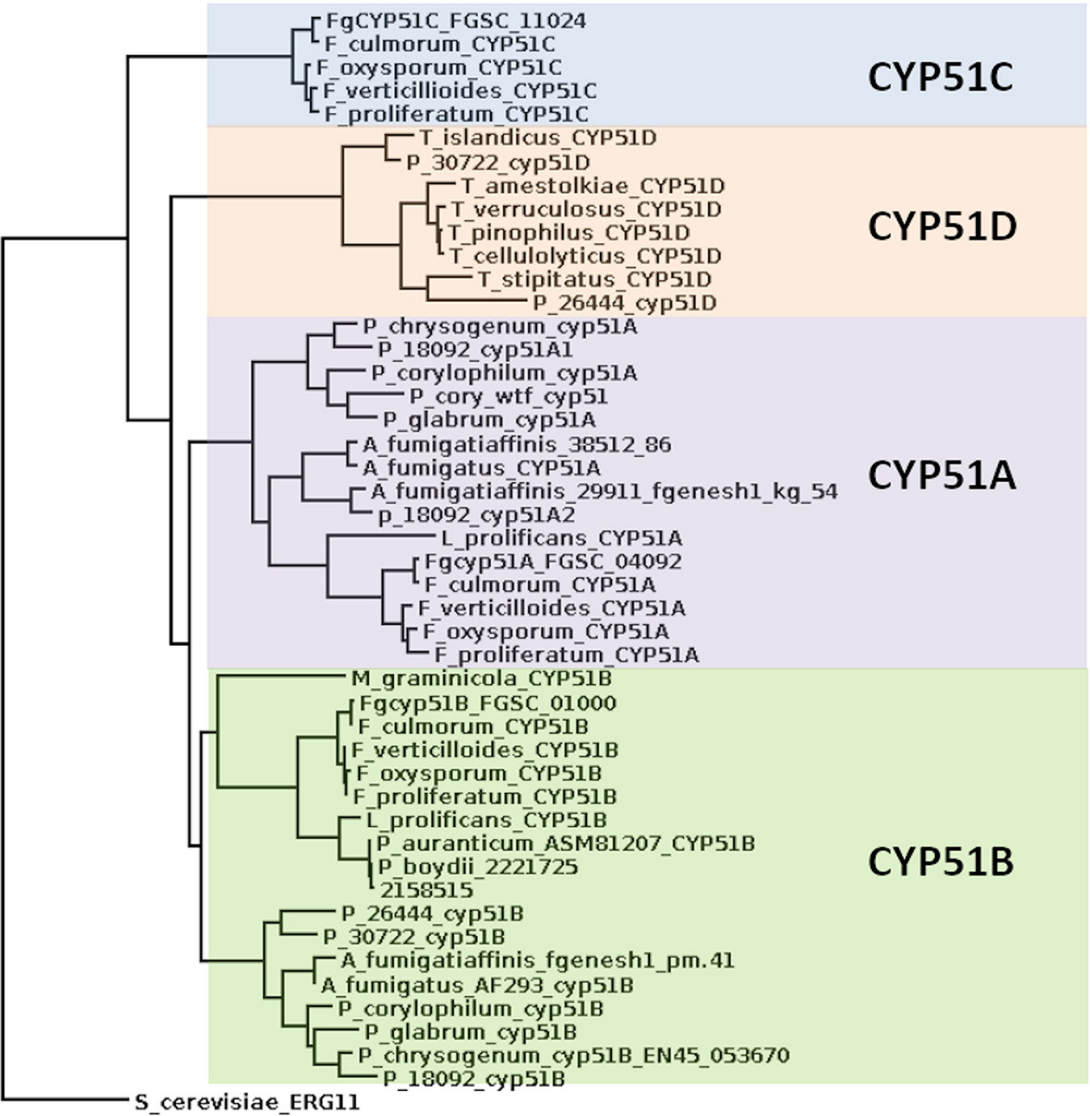
Illustrative phylogeny of CYP51 paralogues in this study. Typical CYP51 paralogues were selected. Complete protein sequences were aligned using MUSCLE, and phylogenies were calculated using FastTree with -gamma -spr 4 -mlacc 2 -slownni parameters.

CYP51 paralogues were identified in the 5 test genomes (Table 1; see also Table S1 in the supplemental material). Surprisingly, the expected dual paralogue CYP51A-CYP51B pattern observed in previously studied Aspergillus and *Penicillium* genomes was observed only in the azole-sensitive Penicillium glabrum isolate. The azole-resistant Penicillium olsonii isolate (Pen spp. 18092) had 2 CYP51A (CYP51A1 and CYP51A2) and one CYP51B paralogues (Fig. 1, Table 1), and the 2 azole-resistant *Talaromyces* species (T. diversus and T. radicus) had no CYP51A paralogue but instead one CYP51B paralogue and a previously undescribed CYP51 paralogue more similar to CYP51B than to the Fusarium CYP51C, which we have termed CYP51D. Finally, the azole-resistant Penicillium corylophilum isolate contained the expected CYP51A and B paralogues but also a novel partial CYP51A gene (CYP51A-wtf [with truncated form]) corresponding to position 275 to C terminus of AFCYP51A). Promoters were checked for the presence of tandem repeats, but none were found.

**TABLE 1:** CYP51 paralogue distribution in fungal species^*a*^

Strain	CYP51A1	CYP51A2	CYP51B	CYP51D	CYP51wtf	Azole res.
T. atroroseus IBT 11181	−	−	+	−	−	?
T. pinophilus AR155	−	−	+	+	−	?
*T. amestolkiae* strain CIB	−	−	+	+	−	I
*T. borbonicus* CBS 141340	−	−	+	+	−	I
A. lentulus CBS 117885	+	−	+	−	−	R
*P. corylophilum* 23421	+	−	+	−	+	R
Pen spp. 18092 (*P. olsonii*)	+	+	+	−	−	R
Pen spp. 26444 (*T. diversus*)	−	−	+	+	−	R
Pen spp. 30722 (*T. radicus*)	−	−	+	+	−	R
*T. aculeatus* ATCC 10409	−	−	+	+	−	R
*T. proteolyticus* PMI_201	−	−	+	+	−	R
*T. stipitatus* ATCC 10500	−	−	+	+	−	R
*Lomentospora prolificans*	+	−	+	−	−	R
Pseudallescheria boydii	−	−	+	−	−	R
Pseudallescheria apiospermum	−	−	+	−	−	R
Pseudallescheria dehoogii	−	−	+	−	−	R
Pseudallescheria aurantiacum	−	−	+	−	−	R
A. fumigatiaffinis	+	+	+	−	−	R
A. fumisynnematus	+	−	+	−	−	S
A. fischeri NRRL 181	+	−	+	−	−	S
A. fumigatus A1163	+	−	+	−	−	S
A. fumigatus AF293	+	−	+	−	−	S
A. fumigatus Z5	+	−	+	−	−	S
Neosartorya hiratsukae CBS 294.93	+	−	+	−	−	S
*P. expansum* MD8	+	−	+	−	−	S
P. antarticum	+	−	+	−	−	S
P. arizonense	+	−	+	−	−	S
P. atramentosum RS17	+	−	+	−	−	S
P. biforme FM169	+	−	+	−	−	S
P. brasilianum ZJ-7	+	−	+	−	−	S
*P. brasilianum*	+	−	+	−	−	S
*P. brasilianum* LaBioMMi136	+	−	+	−	−	S
P. camemberti FM013	+	−	+	−	−	S
P. capsulatum ATCC 48735	+	−	+	−	−	S
*P. capsulatum* LiaoWQ-2-011	+	−	+	−	−	S
P. carnescens ATCC 10419	+	−	+	−	−	S
P. carneum LCP05634	+	−	+	−	−	S
P. chrysogenum 1B	+	−	+	−	−	S
P. chrysogenum HK F42	+	−	+	−	−	S
P. chrysogenum KF25	+	−	+	−	−	S
P. chrysogenum NCPC 10086	+	−	+	−	−	S
P. chrysogenum P2niaD18	+	−	+	−	−	S
P. citrinum	+	−	+	−	−	S
*P. citrinum* DSM 1997	+	−	+	−	−	S
P. coprophilum IBT 31321	+	−	+	−	−	S
P. decumbens IBT 11843	−	−	+	−	−	S
P. digitatum Pd1	+	−	+	−	−	S
*P. digitatum* Pd1 ZJU	+	−	+	−	−	S
*P. digitatum* PDC 102	+	−	+	−	−	S
*P. digitatum* PHI 26	+	−	+	−	−	S
*P. expansum* d1	+	−	+	−	−	S
*P. expansum* NRRL 62431	+	−	+	−	−	S
*P. expansum* R19	+	−	+	−	−	S
*P. expansum* T 01	+	−	+	−	−	S
*P. expansum* YT02	−	−	+	−	−	S
P. glabrum 23851	+	−	+	−	−	S
P. griseofulvum PG3	+	−	+	−	−	S
P. italicum PHI1	+	−	+	−	−	S
P. polonicum IBT 4502	+	−	+	−	−	S
P. raistrickii ATCC 10490	+	−	+	−	−	S
P. solitum IBT 29525	+	−	+	−	−	S
P. swiecickii 182 6C1	+	−	+	−	−	S
P. thymicola DAOMC 180753	+	−	+	−	−	S
*P. expansum* CMP1	+	−	+	−	−	S
T. marneffei ATCC 18224	−	−	+	−	−	S
T. purpureogenus MYA-38	+	−	+	−	−	S

a Paralogues were assigned in each species according to their position in the phylogeny. Resistance for the species was assigned for MICs in the current study or previous literature. *Penicillium* isolates identified in this study are shaded in gray and are single isolates. Symbols and abbreviations: +, paralogue present in the genome; −, paralogue absent in the genome; res., resistance; R, all reported isolates from the species or the average MIC for the species shows azole resistance (>MIC breakpoint for either itraconazole, voriconazole, or posaconazole); I, intermediate resistance observed in the species;?, azole resistance not tested in the species; S, no resistance observed in the species.

10.1128/mBio.01945-21.2 TABLE S1 Overview of sequencing results and CYP51 paralogy determination. Download Table S1, XLSX file, 0.02 MB. Copyright © 2021 Van Rhijn et al. 2021 Van Rhijn et al. https://creativecommons.org/licenses/by/4.0/ This content is distributed under the terms of the Creative Commons Attribution 4.0 International license.

The strains with both CYP51B and -D paralogues (CYP51B-D) were azole resistant, and this suggested that a more general analysis of CYP51 paralogues in other sequenced fungal strains might be fruitful in uncovering potential mechanisms of drug resistance. Representative genomes were selected with a focus on known clinical intrinsically resistant species (Table 1). Paralogues were assigned in each genome as described and checked for correct 1-to-1 orthology by reverse BLAST. The CYP51B-D paralogue structure was observed in a subgroup of *Talaromyces* species (T. stipitatus ATCC 10500, T. borbonicus CBS 141340, T. aculeatus ATCC 10409, T. proteolyticus PMI_201, and T. amestolkiae strain CIB) also noted to be intrinsically azole resistant. A CYP51A, A2, and B paralogue pattern was observed in the azole-resistant species Aspergillus fumigatiaffinis. We note that the intrinsically resistant Aspergillus lentulus has a normal CYP51A-B paralogy pattern. In comparison all Fusarium spp. analyzed had the CYP51A-B-C paralogue structure previously reported.

The nature and mechanisms underpinning intrinsic azole resistance in molds are unknown. Here, we show the existence of a new CYP51 paralogue, CYP51D, which predominantly occurs in genomes of intrinsically azole-resistant Eurotiomycetes. Although we did not observe a perfect correlation between paralogue pattern and intrinsic resistance, this would be expected as many other genomically encoded factors are known to contribute to azole tolerance. The observed correlation was striking with 5/8 species carrying the CYP51B-D paralogue structure being azole resistant, 2/8 displaying intermediate sensitivity, and 1/8 having unknown sensitivity. All 46/46 genome-sequenced azole-sensitive isolates examined in this study had the CYP51A-B paralogue structure; however, 2 intrinsically resistant species, Lomentospora prolificans and A. lentulus, also had a CYP51A-B paralogue structure. Intrinsically resistant *Pseudallescheria* species carried a single CYP51B paralogue.

### Transfer of the CYP51A2 paralogue from A. fumigatiaffinis to Aspergillus fumigatus confers itraconazole resistance.

**FIG 2:**
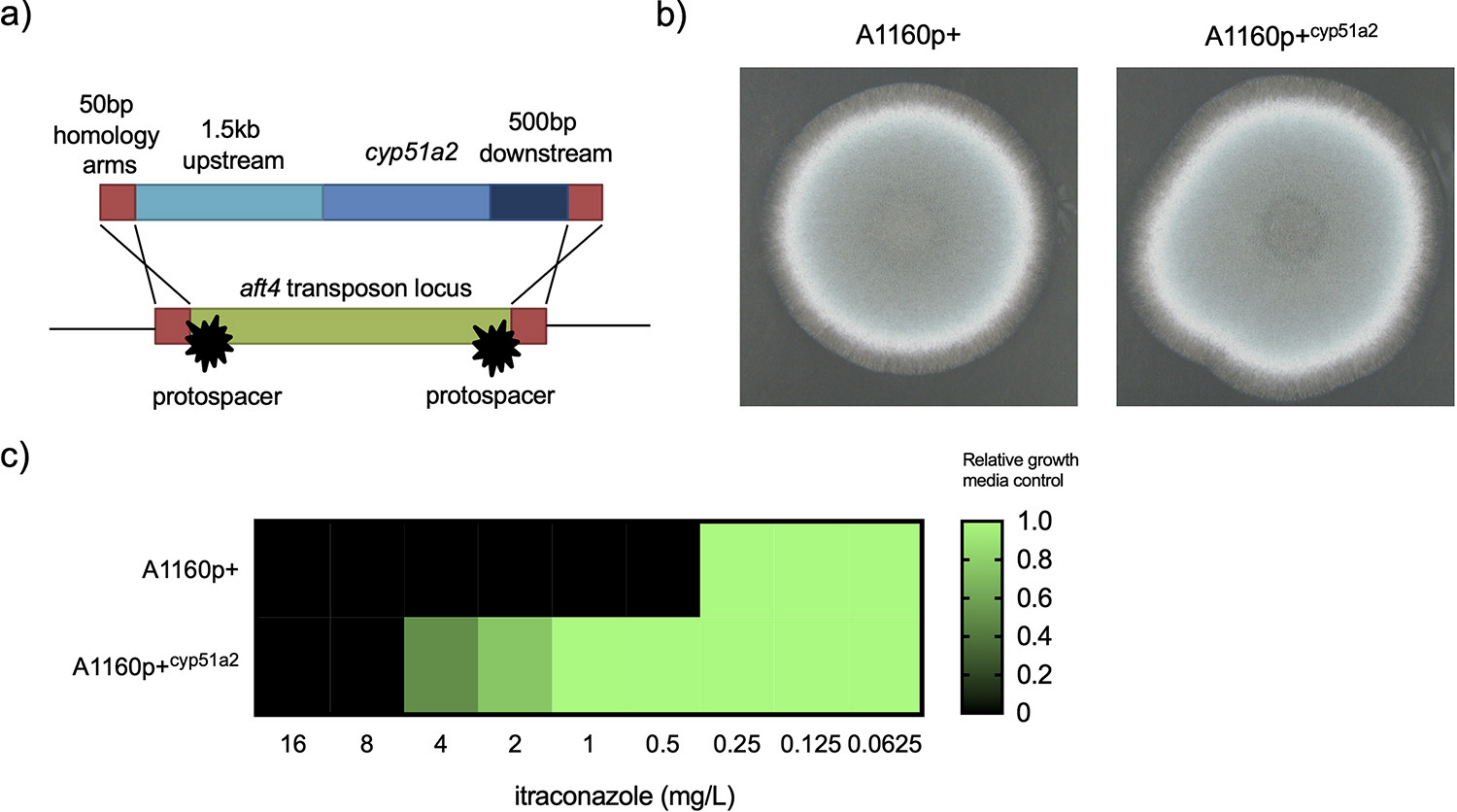
A. fumigatiaffinis *cyp51a2* knock-in results in resistance. (a) Overview of replacing the nonfunctional *aft4* transposon locus with the A. fumigatiaffinis *cyp51a2* gene including 1.5 kb upstream and 500 bp downstream. Microhomology arms of 50 bp are included to allow CRISPR-Cas9-mediated transformation. (b) A phenotypic test on Aspergillus complete medium, grown for 48 h at 37°C. (c) MIC assay for itraconazole for both the parental isolate (A1160p+) and A1160p+^cyp51a2^. Growth was normalized to nondrug control (1% dimethyl sulfoxide [DMSO]).

Two species, A. fumigatiaffinis and *P. olsonii*, had a CYP51A1-A2-B paralogue structure with one species known to be intrinsically resistant and the other noted as resistant in this study. In order to determine the relation between CYP51A2 and azole resistance, we transformed the A. fumigatiaffinis CYP51A2 into A. fumigatus A1160p+ (Fig. 2a, Fig. S1, and Table S2). While no apparent defects in radial growth or morphology were observed in the transformant (Fig. 2b), A1160p+^cyp51a2^ was resistant to itraconazole (8 mg/liter) compared to the parental isolate (0.5 mg/liter) (Fig. 2c). MICs observed for this A. fumigatus strain are comparable to the MIC determined for A. fumigatiaffinis isolates (11). The presence of two paralogous, nonidentical CYP51A genes could potentially explain the observed azole resistance in A. fumigatiaffinis.

10.1128/mBio.01945-21.1 FIG S1 Validation of *cyp521a2* insertion into the *aft4* locus. Overview of the PCR strategy to validation targeted insertion of the *cyp51a2* locus and result. Download FIG S1, TIF file, 0.4 MB. Copyright © 2021 Van Rhijn et al. 2021 Van Rhijn et al. https://creativecommons.org/licenses/by/4.0/ This content is distributed under the terms of the Creative Commons Attribution 4.0 International license.

10.1128/mBio.01945-21.3 TABLE S2 Primer sequences. Download Table S2, XLSX file, 0.01 MB. Copyright © 2021 Van Rhijn et al. 2021 Van Rhijn et al. https://creativecommons.org/licenses/by/4.0/ This content is distributed under the terms of the Creative Commons Attribution 4.0 International license.

It should be stressed that the current study shows only a correlation between the presence of CYP51D and azole resistance (*P* < 0.0001 assuming random selection of genomes) but that no causal relationship has been tested for CYP51D. The Fusarium CYP51C paralogue does not appear to play a role in either azole resistance or ergosterol biosynthesis; however, CYP51D is unrelated to CYP51C and groups more closely with CYP51B (Fig. 1). This may support a role for CYP51D in ergosterol biosynthesis, but considerable further work is required to confirm this hypothesis. Additionally, the analysis presented here could be limited by the availability of genome sequences which may be biased toward “interesting” genomes displaying phenotypes such as drug insensitivity. Perusal of the available genomes does not support this, with most arising from projects aimed at use of certain species in food or antibiotic production or primarily as pathogens with no focus on drug sensitivity. There is little depth in the literature describing azole resistance in many of the species included in this paper, and it would be desirable to perform a survey of multiple representatives of each species to properly assess MICs for several azoles to support these findings. Finally, our focus on CYP51D does not preclude the possibility that lack of the CYP51A paralogue in CYP51B-D paralogue genomes may also affect azole resistance as this is potentially the primary target of azoles. Isolates carrying the CYP51B-D paralogues grew in a normal manner and exhibited abundant sporulation, so presumably they are able to synthesize adequate sterol membrane components.

The observation that transfer of a CYP51A2 paralogue from a resistant species into a sensitive species results in azole resistance suggests that intrinsic azole resistance can arise from CYP51 paralogue structure, and this observation, together with an accurate understanding of paralogue number and type, will provide an important guide to understanding intrinsic azole insensitivity or tolerance in fungal species. We note that the CYP51A2 allele from A. fumigatiaffinis does not have altered amino acids at positions previously identified as being important for resistance in the A. fumigatus CYP51A gene, such as L98, G54, or M220. This may mean that higher protein levels resulting from expression of 2 gene copies rather than failure of drug to bind protein could explain the altered azole sensitivity. Further confirmation of such observations, for example, by deletion of paralogues in intrinsically resistant species, awaits development of appropriate transformation methodology in these species.

### Experimental procedures.

Five *Penicillium* species isolates were obtained from the NHS Mycology Reference Centre, Manchester, United Kingdom. The isolates had been tentatively identified as *Penicillium* spp. by morphology, and azole resistance was determined by MICs for itraconazole, voriconazole, and posaconazole using EUCAST procedures (12).

### (i) Genome sequencing.

DNA was prepared as previously described for Aspergillus fumigatus (4) and sequenced with a paired-end protocol using an Illumina HiSEQ2000. Genomes were assembled using Velvet (13) with a 31-bp kmer input and queried using BLASTN and TBLASTN (14) and a set of Aspergillus fumigatus, Fusarium graminearum, and Penicillium chrysogenum CYP51 query protein sequences (Fgcyp51A; FGSC_04092, Fgcyp51B; FGSC_01000, FgCYP51C; FGSC_11024, P. chrysogenum CYP51A; EN45_094440, P. chrysogenum CYP51B; EN45_053670). Orthology and paralogy were confirmed by BLAST back to the original reference genomes. Genomes for comparison were obtained from NCBI (accessed May 2020) and JGI databases (15, 16), and coordinates for each CYP51 paralogue were compared (Table S1). Sequences for β-tubulin and the RNA polymerase subunits RPB1 and −2 were also extracted using TBLASTN with query sequences derived from P. chrysogenum and used for species identification. BLAST analysis was handled using bash scripted pipelines.

Predicted CYP51 protein sequences were aligned using MUSCLE, manually checked and corrected, and then trimmed to remove unconserved N-terminal residues before phylogenetic tree construction (Fig. 1). Data on azole sensitivity and phylogenies for *Penicillium* and *Talaromyces* were obtained from previous publications (17–21).

Genome sequencing and assembly resulted in *N*_50_ of >80 kb and coverage of >75× for all genomes. Genomes and genome assemblies from public databases all also showed similar or better assembly profiles.

### (ii) Genome-based identification of isolates.

The 5 *Penicillium* isolates were identified using β-tubulin, RPB1, and RPB2 species-level identification markers as previously defined (21). Protein sequences derived from the isolate genome sequence assembly compared to publicly available genome sequences confirmed the identity of strain 23421 as *P. corylophilum* (100% protein identity [ID] to genomic *P. corylophilum* β-tubulin, RPB1 and RPB2 genes) and 23851 as P. glabrum (99, 99, and 100% ID, respectively). *Penicillium* species 30722 was identified as *Talaromyces radicus* (99, 100, and 100% ID, respectively), *Penicillium* species 26444 was identified as *Talaromyces diversus* (99, 100, and 100% ID, respectively), and *Penicillium* species 18092 was identified as *P. olsonii* (99, 100, and 100% ID, respectively).

### (iii) CYP51A2 transfer into A. fumigatus.

The CYP51A2 gene was amplified from A. fumigatiaffinis genomic DNA using primers cyp51a2_Fw and cyp51a2_Rv (Table S2) including 1.5 kb upstream and 500 bp downstream of the predicted open reading frame (ORF), followed by gel purification (NucleoSpin; Macherey-Nagel). CYP51A2 was transformed into a genomic “safe haven” in A. fumigatus A1160p+ using selection-free CRISPR-Cas9-mediated transformation with crRNA atf4_TIRrm_up and aft4_TIRrm_down (22, 23). Transformants were purified twice on Sabouraud agar and PCR verified using cyp51a2_chk_fw and aft4_screening_rv. A phenotypic assay was performed by spotting 1,000 spores in 5 μl onto Aspergillus complete medium (ACM) incubated for 48 h at 37°C. MIC assays for itraconazole were performed according to EUCAST methodology (12).
