# Supplementary figures and images for: CYP51 Paralogue Structure Is Associated with Intrinsic Azole Resistance in Fungi
Source: mBio. 2021 Oct 5;12(5):e01945-21. doi: 10.1128/mBio.01945-21 (PMC8546618; doi:10.1128/mBio.01945-21)

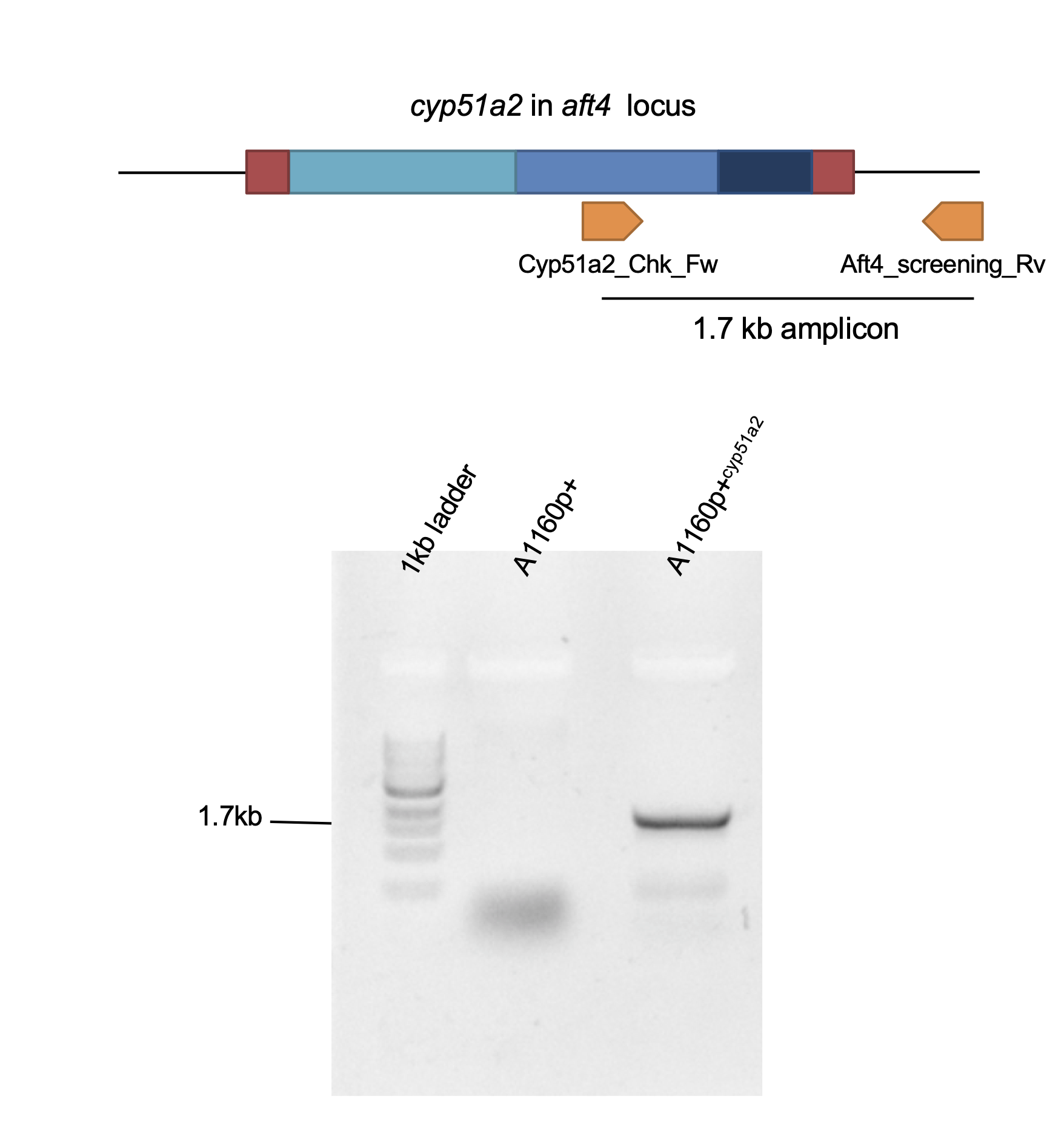

Supplement: FIG S1 [file mbio.01945-21-sf001.tif]
